# A 61-year-old man from Iran with intermittent obstruction of colon 

**Published:** 2021

**Authors:** Elahe Zanganeh, Seyed Ashkan Hosseini, Mehdi Alimadadi, Mohammadreza Seyyedmajidi

**Affiliations:** 1 *Golestan Research Center of Gastroenterology and Hepatology (GRCGH), Golestan University of Medical Sciences, Gorgan, Iran*; 2 *Mashhad University of Medical Sciences, Mashhad, Iran*

## Introduction

 A 61-year-old, otherwise healthy man presented to our hospital complaining of intermittent colicky pain of left-sided abdomen, bloating, and constipation for the prior month. He had no abdominal surgeries or significant problems in his past medical history. Physical examination revealed mild anemia and abdominal tenderness. The patient’s hemoglobin level was 11.8 g/dL and his carcinoembryonic antigen level (CEA), erythrocyte sedimentation rate (ESR), and C-reactive protein (CRP) were normal.

**Figure 1:**
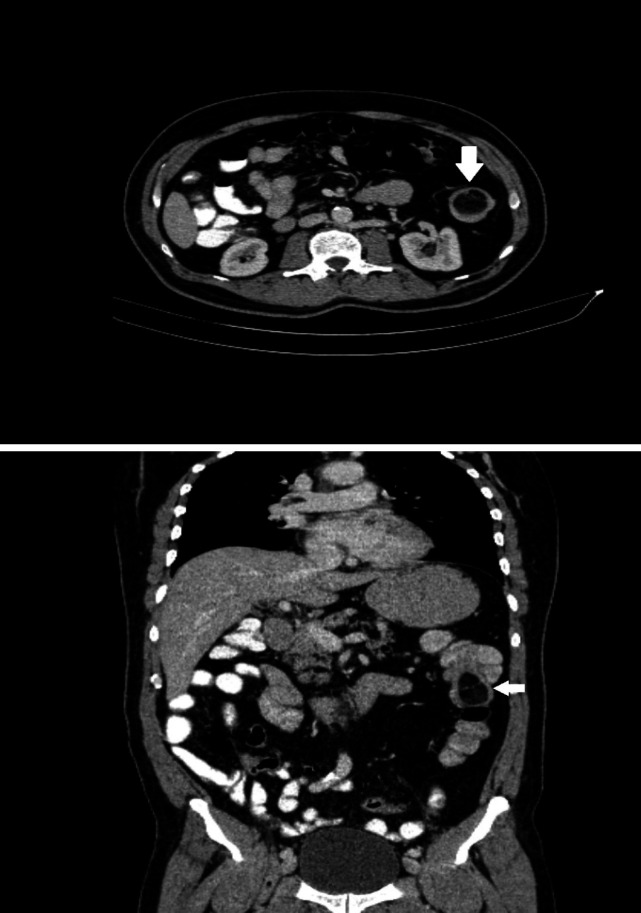
A mass on the descending colon (white arrow) with a density of approximately -100 Hounsfield units on computed tomography (CT)

**Figure 2:**
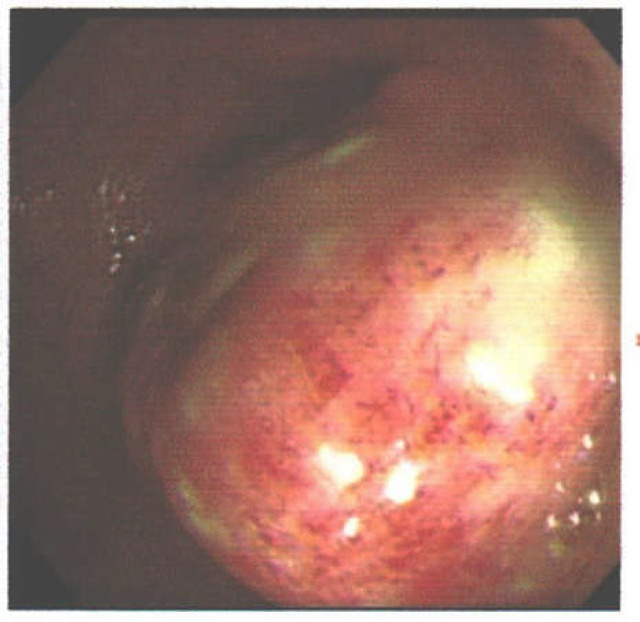
A giant submucosal mass at the descending colon in colonoscopy

Contrast-enhanced abdominal computed tomography (CT) revealed the presence of a concentric mass in the descending colon with a density of approximately -100 Hounsfield units (Figure 1). A colonoscopy was performed for a better assessment, which revealed a giant submucosal mass on the descending colon that encompassed more than 75% of the bowel lumen (Figure 2). A biopsy was performed and a histopathologic examination of the specimens showed reactive changes without evidence of dysplasia or malignancy, but a definite diagnosis could not be made. The patient underwent a segmental colonic resection by laparotomy because of the size of the mass and the inability to rule out a malignancy. 

**What is your diagnosis?**

**Answer:** Descending colon lipoma. Pathological examination of the surgical specimen showed mature adipocytes in the submucosa with mucosal ulceration with no signs of malignancy.

## Discussion

Lipomas of the gastrointestinal tract are rare non-epithelial tumors and most often discovered incidentally during colonoscopy, imaging, surgery, or autopsy. Colonic lipomas are more prevalent in women and have a peak incidence in patients between the 4th and 7th decade of life; their incidence ranges from 0.2% to 4.4%. Common sites of colonic lipomas are the ascending colon (45%), sigmoid colon (30%), descending colon (15%), and transverse colon (9%), respectively (1, 2). 

These tumors are rarely greater than 2 cm in size and are usually asymptomatic. If they exceed 2 cm, however, they may cause different symptoms such as abdominal pain, change in bowel habits, bleeding, intussusception, or obstruction (3). They are often difficult to diagnose because of their asymptomatic nature or the intermittent symptoms. A definitive diagnosis can be made after the lipoma is removed and subjected to histopathologic staining. Surgical resection is recommended to alleviate symptoms and to rule out malignancy (4). Many cases of lipomas with overlying villous adenomas or other presentations mimicking carcinomas have been reported (5). 

They are rare neoplasms of mesenchymal origin presenting as a polypoid tumor that protrudes into the lumen (6). 90% of them are localized to the submucosa and rarely found in other layers of the bowel wall (7). Chronic inflammation may play a role in the pathogenesis through a mechanism of increased intestinal motility that causes consequent detachment of the mucosa (8). These tumors have a dimension extending from 2 cm to 30 cm. They are usually solitary, but they can also be multiple (9).

Contrast-enhanced abdominal computed tomography (CT) is a valuable non-invasive imaging method for the diagnosis of colonic lipomas. The characteristic sign is an intraluminal mass with regular, ovoid, and sharp borders at a homogeneous fat density. In MRI, the signal intensity of adipose tissue and suppression of signal in fat-suppressed sequences have been seen; therefore, MRI may be especially beneficial in the detection of lipomas (10).

Colonoscopy is suggested when a diagnosis of colonic cancer is suspected. Three endoscopic signs can aid: (a) the “cushion” sign, which occurs when forceps press into the mass, resulting in pillowing; (b) the “tenting” sign, which occurs when mucosa is grabbed and pulled away, resulting in a tent-like appearance; (c) the “naked fat” sign, which occurs when fat is extruded after biopsy (1). In some cases, endoscopy may reveal ulcerative lesions of the polypoid mass leading to a presumption of malignancy. Endoscopic ultrasonography can help demonstrate the presence of a hyperechoic mass. Evaluation of the tumor’s base is the key of indication for endoscopic resection. There are several techniques for endoscopic removal, including endo-loop, nylon loop, endo-clip resection, and sectioning of the overlying mucosa via segmental cuts (11).

Surgery is the standard therapeutic option for lipomas with a diameter greater than 2 cm. It may also be warranted in cases of unclear preoperative diagnosis, symptomatic patient, involvement of the muscular or serosal layer, and when the lesion cannot be radically resected endoscopically (1). Laparoscopic resection under colonoscopic guidance of lipomas of the colon has been also reported (12).

## Conflict of interests

The authors declare that they have no conflict of interest.
